# Long‐term outcomes of rapid antiretroviral NNRTI‐based initiation among Thai youth living with HIV: a national registry database study

**DOI:** 10.1002/jia2.26071

**Published:** 2023-03-21

**Authors:** Sirinya Teeraananchai, Stephen J. Kerr, Kiat Ruxrungtham, Panthep Khananuraksa, Thanyawee Puthanakit

**Affiliations:** ^1^ Department of Statistics Faculty of Science Kasetsart University Bangkok Thailand; ^2^ HIV‐NAT Thai Red Cross AIDS Research Centre Bangkok Thailand; ^3^ Biostatistics Excellence Centre Faculty of Medicine Chulalongkorn University Bangkok Thailand; ^4^ Kirby Institute University of New South Wales Sydney New South Wales Australia; ^5^ Department of Medicine Faculty of Medicine Chulalongkorn University Bangkok Thailand; ^6^ Chula Vaccine Research Center (ChulaVRC) School of Global Health Faculty of Medicine Chulalongkorn University Bangkok Thailand; ^7^ National Health Security Office Bangkok Thailand; ^8^ Division of Infectious Diseases Department of Pediatrics Faculty of Medicine Chulalongkorn University Bangkok Thailand; ^9^ Center of Excellence in Pediatric Infectious Diseases and Vaccines Chulalongkorn University Bangkok Thailand

**Keywords:** youth living with HIV, antiretroviral therapy, rapid ART, virological failure, second‐line regimen, cohort studies

## Abstract

**Introduction:**

The Thai National AIDS programme (NAP) treatment guidelines have recommended rapid antiretroviral therapy (ART) initiation, regardless of CD4 count since 2014. We assessed treatment outcomes among youth living with HIV (YLHIV), initiating first‐line ART and assessed the association between virological failure (VF) and timing of ART initiation.

**Methods:**

We retrospectively reviewed data for YLHIV aged 15–24 years, initiating non‐nucleoside reverse transcriptase inhibitor‐based ART from 2014 to 2019, through the NAP database. We classified the timing of ART into three groups based on duration from HIV‐positive diagnosis or system registration to ART initiation: (1) <1 month (rapid ART); (2) 1–3 months (intermediate ART); and (3) >3 months (delayed ART). VF was defined as viral load (VL) ≥ 1000 copies/ml after at least 6 months of first‐line ART. Factors associated with VF were analysed using generalized estimating equations.

**Results:**

Of 19,825 YLHIV who started ART, 78% were male. Median (interquartile range, IQR) age was 21 (20–23) years and CD4 count was 338 (187–498) cells/mm^3^. After registration, 12,216 (62%) started rapid ART, 4272 (22%) intermediate ART and 3337 (17%) delayed ART. The proportion of YLHIV starting ART <30 days significantly increased from 43% to 57% from 2014–2016 to 2017–2019 (*p* < 0.001). The median duration of first‐line therapy was 2 (IQR 1–3) years and 89% started with efavirenz‐based regimens. Attrition outcomes showed that 325 (2%) died (0.73 [95% CI 0.65–0.81] per 100 person‐years [PY]) and 1762 (9%) were loss to follow‐up (3.96 [95% CI 3.78–4.15] per 100 PY). Of 17,512 (88%) who had VL checked from 6 to 12 months after starting treatment, 80% achieved VL <200 copies/ml. Overall, 2512 experienced VF 5.87 (95% CI 5.65–6.11) per 100 PY). In a multivariate model, the adjusted incidence rate ratio for VF was 1.47 (95% CI 1.33–1.63, *p* < 0.001) in the delayed ART group and 1.14 (95% CI 1.03–1.25, *p*< 0.001) in the intermediate ART group, compared to YLHIV in the rapid ART group.

**Conclusions:**

Rapid ART initiation after diagnosis was associated with significantly reduced risks of VF and death in YLHIV, supporting the implementation of rapid ART for optimizing health outcomes.

## 1 INTRODUCTION

In 2015, the World Health Organization (WHO) recommended commencing antiretroviral therapy (ART) at any CD4 count [1]. As countries have adopted this “treat all” policy, the time to initiate ART after an HIV diagnosis has decreased, and the proportion of people living with HIV (PLHIV) who rapidly initiate ART rapidly has increased [2, 3, 4]. Several studies reported that rapid ART initiation is associated with reduced morbidity and mortality, and a higher probability virological suppression and retention in care [5, 6, 7, 8]. WHO subsequently recommended initiating treatment within 7 days of a confirmed HIV diagnosis, and same‐day initiation when feasible [9].

Adoption of treat all policies has led to increasing rates of same‐day ART, or rapid ART initiation within 30 days of diagnosis [2, 5, 6, 10]. However, even in these contexts, treatment outcomes may still fall short of WHO 95:95:95 goals. A same‐day ART implementation science project in adults in Bangkok in 2017/2018 demonstrated retention rates of 75.3%, only 53.4% received viral load (VL) testing, and only 49.3% of all participants initiating ART achieved viral suppression within the first year [8]. In the era of rapid ART initiation, the largest group of new HIV diagnoses are youth who acquired HIV behaviourally, particularly young men who have sex with men [11, 12, 13]. Before “treat all” policies came into effect, in many different settings, these youth had loss to follow‐up (LTFU) rates ranging from 11% to 48% prior ART initiation. These LTFU rates were substantially higher than rates in adults, which ranged from 10.8% to 38% [14, 15, 16, 17, 18]. Other studies have shown reductions in LTFU among those >18 years after the implementation of rapid ART policies, and youth living with HIV (YLHIV) aged between 15 and 24 years were at higher risk of attrition compared to adults living with HIV [19, 20, 21, 22, 23, 24].

In Thailand, PLHIV are offered free HIV care and treatment through the National AIDS programme (NAP) [25] and national treatment guidelines are regularly updated based on the best available evidence, to optimize treatment outcomes [26]. In a recent study of treat all policy on rapid ART initiation in Thai YLHIV, the proportion initiating ART within 1 month after registration increased from 27% in the year 2014 to 52% in 2019, and ART initiation within 6 months after registration increased from 47% to 74% [27]. However, long‐term outcomes data in youth by timing of ART initiation in real‐world settings are limited. We aimed to assess long‐term treatment outcomes among YLHIV, initiating first‐line, non‐nucleoside reverse transcriptase inhibitor (NNRTI)‐based ART in the NAP, and assessed the association between virological failure (VF) and timing of ART initiation. We also determined factors associated with switches to second‐line regimens.

## 2 METHODS

### 2.1 The NAP database

The NAP programme is the main health insurance programme in Thailand for delivering treatment and care to PLHIV [25, 27]. Hospitals and clinical sites enter laboratory results, clinical information and ART dispensing records into the database system at patient registration and subsequent visits; this information is used to reimburse hospitals for laboratory tests and medications provided. CD4 tests are conducted every 6 months, and HIV‐RNA is measured 6 months after starting ART, and annually thereafter. ART is provided based on National HIV treatment guidelines [26]. The database is linked with the National Death Registry, and vital status is updated daily.

### 2.2 Study population

This retrospective cohort study included YLHIV aged 15–24 years, who initiated treatment with National Guideline recommended NNRTI‐based ART between January 2014 and May 2019. Follow‐up data were available until May 2020. YLHIV were eligible for inclusion if they had at least one VL test after ART initiation during follow‐up for treatment monitoring. We allowed at least a year of follow‐up for all patients to establish this treatment outcome. We assumed the majority of YLHIV acquired HIV behaviourally, since HIV transmission mode is not recorded in the database; our assumption reflects the situation in Thailand during the study period [12]. Patients who started with nucleoside reverse‐transcriptase inhibitors only or protease inhibitors (PIs) were excluded; guidelines recommend PI‐based ART for pregnant women, but we were unable to ascertain pregnancy status [26]. Integrase strand transfer inhibitor‐based regimens were not recommended at the time of the study, and therefore, not available for use in the NAP. We excluded patients who initiated ART before NAP enrolment, because no clinical information for these patients was available at ART initiation. Only 5% of PLHIV started treatment in facilities outside the NAP [25]. Patients taking ART <6 months were also excluded since no VL tests are done during this period. This study was approved by the Institutional Review Board of the Institute for Development of Human Research Protection, Ministry of Public Health, Thailand. A waiver of consent was granted for this data analysis. The NAP database was de‐identified by the National Health Security Office before analysis.

### 2.3 Definitions and outcomes

We classified YLHIV into three groups based on duration from registration (HIV‐positive test) to ART initiation following treatment guidelines for 2014–2019. Those starting ART <1 month from registration were classified as rapid ART. Intermediate ART was defined as starting ART 1–3 months after registration; those starting >3 months were defined as delayed ART. Periods of starting ART were classified into two periods (2014–2016 and 2017–2019) to assess trends in accessing rapid ART. NNRTI‐based treatment included NNRTIs available in Thailand, namely efavirenz (EFV), nevirapine (NVP) or rilpivirine (RPV). VL within the first year was defined as VL measurements from 6 to 12 months after ART initiation. VF was defined as VL ≥ 1000 copies/ml during follow‐up. Follow‐up time on first‐line therapy was censored on the date of the last visit or the date of switching to second‐line ART for those who switched. Patients who died or were LTFU were censored at their last visit. Switching to second‐line ART was defined as changing from an NNRTI‐ to PI‐based regimen, with confirmed VF after at least 6 months of treatment. Mortality was confirmed in the death registry. LTFU was defined as not attending clinic visits for ≥ 12 months, irrespective of whether or not patients later returned to NAP after ART initiation. Pre‐ART CD4 count was defined as the closest result within a window 12 months before, or up to 1 month after the date of ART initiation. Geographic regions of Thailand were classified according to classifications used by government organizations. History of opportunistic infections at ART initiation was entered in the database as “yes or no.”

### 2.4 Statistical analysis

Baseline demographic, disease and treatment information were summarized by ART initiation group. Comparisons of categorical characteristics between ART groups were performed using Pearson's Chi‐square test and continuous characteristics were compared using a Kruskal–Wallis test. Mortality, LTFU, VF and second‐line ART switch rates were calculated per 100 person‐years of follow‐up (PY). Associations between timing of ART initiation and VF were analysed using generalized estimating equations (GEE) model with a Poisson distribution, log link function and exchangeable correlation matrix. Competing risk models [28] were used to calculate the cumulative incidence of second‐line ART switch, and sub‐distribution hazard ratios (SHRs) quantitated associations between patient characteristics and the second‐line switch. LTFU and death were considered competing events. Covariates assessed in both GEE and competing risk models included age, gender, ART initiation group, first regimen, pre‐ART CD4, country region, year of starting ART and opportunistic infections.

Variables with *p* < 0.10 in univariable screening were adjusted for in multivariable models. Statistical significance was identified using a two‐sided *p* value less than 0.05. Statistical analysis was performed with SAS version 9.4 (SAS Institute Inc, Cary, NC) and Stata version 16 (StataCorp, College Station, TX).

## 3 RESULTS

A total of 21,968 youth started ART through the NAP from 2014 to 2019. We excluded 1320 (6%) youth who initiated PI‐based regimens, and 823 (4%) who were not eligible for VL testing because the duration on ART was <6 months. This resulted in an analysis population of 19,825 youth.

### 3.1 Patient characteristics

Table 1 shows the baseline characteristics of the 19,825 YLHIV by ART initiation group. The date of HIV diagnosis was recorded in 73%; in the remaining YLHIV, the NAP registration date was used as a surrogate for the HIV diagnosis date and used to calculate the time to ART initiation. The median duration from registration to ART initiation was 21 (interquartile range, IQR 6–53) days. The rapid ART group comprised 12,216 (62%) YLHIV (17% same‐day ART, 13% within 7 days, 32% >7 days to 1 month). The intermediate and delayed ART initiation groups comprised 4272 (22%) and 3337 (17%) YLHIV, respectively. The majority of patients were male; the median age at ART initiation was 21 (IQR 20–23) years. Five percent had an opportunistic infection at baseline; the median pre‐ART CD4 count was 338 (IQR 187–498) cells/mm^3^. Most YLHIV started with EFV‐ based regimens (89%), followed by NVP‐ (10%) and the smallest proportion started with RPV‐based ART (1%) which only became available in later years of the study period. The majority of YLHIV (25%) were from the Northeastern region.

**Table 1:** Characteristics of youth living with HIV at ART initiation stratified by the time of ART initiation

	ART initiation group (duration from diagnosis to starting ART)				
Characteristics	Rapid (<1 month)	Intermediate (1–3 months)	Delayed (>3 months)	Total	*p*
***N* (%)**	12,216 (62)	4272 (22)	3337 (17)	19,825	
**Gender, *N* (%)**					
Male	9301 (76)	3483 (82)	2722 (82)	15,506 (78)	<0.001
Female	2915 (24)	789 (18)	615 (18)	4319 (22)	
**Median (IQR) age (years)**	21 (19–23)	21 (20–23)	22 (21–24)	21 (20–23)	<0.001
**Age category**					<0.001
15 to <20 years	3916 (32)	1199 (28)	595 (18)	5710 (29)	
≥20 years	8300 (68)	3073 (72)	2742 (82)	14,115 (71)	
**Pre‐ART CD4 available, *N* (%)**	11,521 (94)	4131 (97)	3094 (93)	18,746 (95)	
**Median (IQR) pre‐ART CD4 count (cells/mm^3^)**	345 (208–501)	271 (96–438)	393 (217–560)	338 (187–498)	<0.001
**First regimen**					<0.001
NVP based	998 (8)	577 (14)	332 (10)	1907 (10)	
EVP based	11,112 (91)	3669 (86)	2946 (88)	17,727 (89)	
RPV based	106 (1)	26 (1)	59 (2)	191 (1)	
**Opportunistic infection at ART initiation**					<0.001
Yes	422 (3)	396 (9)	235 (7)	1053 (5)	
No	11,794 (97)	3876 (91)	3102 (93)	18,772 (95)	
**Year of ART initiation**					<0.001
2014–2016	5274 (43)	2191 (51)	1551 (46)	9016 (45)	
2017–2019	6942 (57)	2081 (49)	1786 (54)	10,809 (55)	
**Region**					<0.001
Bangkok	2472 (20)	555 (13)	707 (21)	3734 (19)	
Central	1990 (16)	760 (18)	520 (16)	3270 (16)	
Northeastern	3115 (26)	1137 (27)	714 (21)	4966 (25)	
Northern	2340 (19)	772 (18)	551 (17)	3663 (18)	
Eastern	1102 (9)	419 (10)	396 (12)	1917 (10)	
Southern	868 (7)	383 (9)	294 (9)	1545 (8)	
Western	329 (3)	246 (6)	155 (5)	730 (4)	

Notes: The comparisons were performed using Pearson's Chi‐square tests, for categorical data, and Kruskal–Wallis tests for continuous data. Presented as *n* (%) for categorical data and median (interquartile range) for continuous data.

Abbreviations: ART, antiretroviral therapy; EFV, efavirenz; NVP, nevirapine; RPV, rilpivirine.

**Figure 1:**
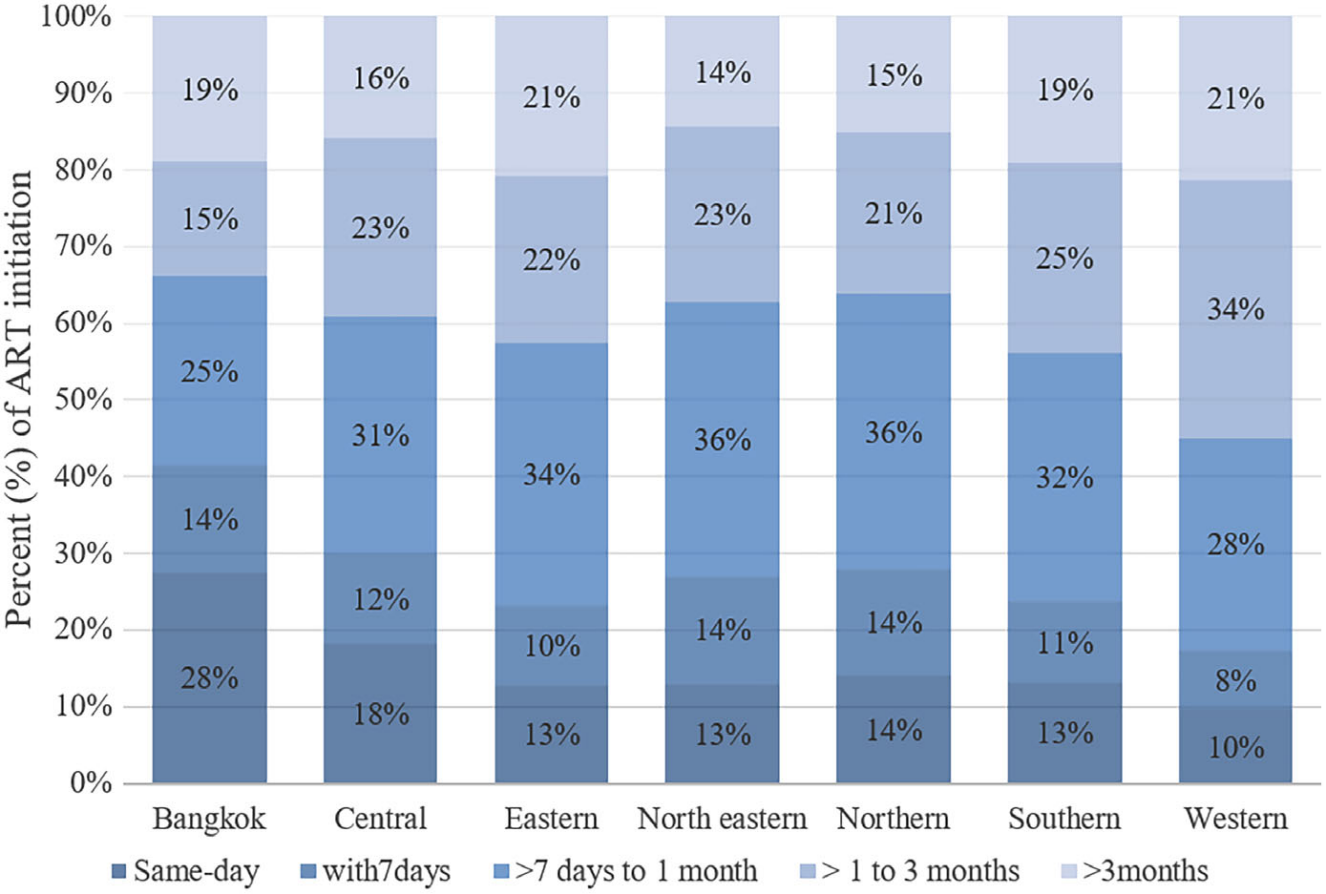
The percentage of ART initiation in youth living with HIV in the Thai National HIV Treatment programme, by timing of ART initiation and regions. Abbreviation: ART, antiretroviral therapy.

Figure 1 shows the percentage of YLHIV starting ART by timing relative to diagnosis and region. YLHIV in Bangkok had the highest percentage starting rapid ART (66%), whereas the Western region had the highest percentages in the intermediate ART (34%) and delayed ART groups (21%). The highest percentage of YLHIV who started same‐day ART (28%) were in Bangkok.

For the ART initiation group, YLHIV who initiated ART within 1 month from HIV diagnosis increased from 43% in 2014–2016 to 57% in 2017–2019, with a median duration from registration to initiation of 2 (IQR 1–3) days. Those initiating same‐day ART and starting ART within 7 days both increased by 3% in the second study period (15–18%, and 11–14%, respectively). Median pre‐ART CD4 count in YLHIV in rapid and delayed ART groups was higher versus the intermediate ART group (345 vs. 393 vs. 271 cells/mm^3^, *p* <0.001). Median pre‐ART CD4 in the rapid ART group increased from 331 (IQR 194–490) to 356 (IQR 221–511) cells/mm^3^ from 2014–2016 to 2017–2019.

### 3.2 Mortality and LTFU outcomes

**Figure 2:**
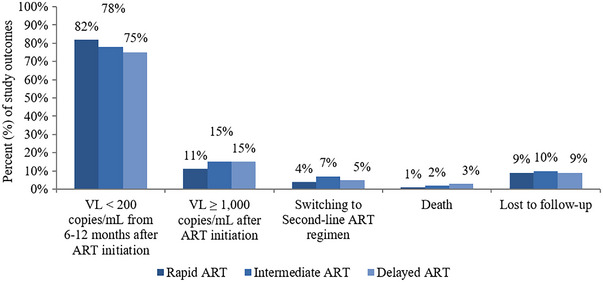
Study outcomes after ART initiation in youth living with HIV in the Thai National HIV Treatment programme, by timing of ART initiation. Notes: The proportion of VL < 200 copies/ml was divided by the number of patients who had VL from 6 to 12 months after ART initiation. There were statistically significant differences in the proportion of youth experiencing all outcomes by ART initiation group (*p* < 0.001). Abbreviation: ART, antiretroviral therapy.

Outcomes after ART initiation are shown in Figure 2. Of 1762 (9%) YLHIV were LTFU with an overall crude LTFU rate was 3.96 (95% CI 3.78–4.15) per 100 PY. YLHIV in the delayed ART group had the highest LTFU rate (4.11 [95% CI 3.68–4.60] per 100 PY), followed by those in intermediate (4.00 [95% CI 4.63–4.41] per 100 PY) and rapid ART groups (3.90 [95% CI 3.67–4.15] per 100 PY, *p* = 0.70). For mortality, 325 (2%) died, giving an overall crude mortality rate of 0.73 (95% CI 0.65–0.81) per 100 PY. Mortality rates increased as the time to initiate ART increased. The mortality rate was the highest in the delayed ART group (1.23 [95% CI 1.00–1.51] per 100 PY) followed by intermediate ART (0.87 [95% CI 0.71–1.07] per 100 PY) and rapid ART group (0.53 [95% CI 0.45–0.62] per 100 PY, *p*<0.001) (Table 2).

**Table 2:** Study outcomes of youth living with HIV by timing of ART initiation

	ART initiation group (duration from diagnosis to starting ART)				
	Rapid (<1 month)	Intermediate (1–3 months)	Delayed (>3 months)	Total	*p*
***N* (%)**	12,216 (62)	4272 (22)	3337 (17)	19,825	
**Person‐year of follow‐up**	26,718	10,267	7491	44,476	
**LTFU after ART initiation, *n* (%)**	1043 (9)	411 (10)	308 (9)	1762 (9)	0.005
**Crude LTFU rate per 100 PY (95% CI)**	3.9 (3.67–4.15)	4 (3.63–4.41)	4.11 (3.68–4.6)	3.96 (3.78–4.15)	0.70
**Person‐year of follow‐up**	26,845	10,357	7569	44,771	
**Death after starting ART, *n* (%)**	149 (1)	93 (2)	91 (3)	333 (2)	<0.001
**Crude death rate per 100 PY (95% CI)**	0.53 (0.45–0.62)	0.87 (0.71–1.07)	1.23 (1–1.51)	0.73 (0.65–0.81)	<0.001
**Viral load from 6 to 12 months after ART initiation measurement, *N* (%)**	10,934 (90)	3755 (88)	2823 (85)	17,512 (88)	
**VL < 50 copies/ml, *N* (%)^a^**	9137 (84)	3035 (81)	2301 (82)	14,473 (83)	<0.001
**VL < 200 copies/ml, *N* (%)^a^**	10,052 (92)	3353 (89)	2503 (89)	15,908 (91)	<0.001
**VF after ART initiation, *n* (%)**	1378 (11)	649 (15)	485 (15)	2512 (13)	<0.001
**Crude VF rate per 100 PY (95% CI)**	5.35 (5.08–5.64)	6.59 (6.11–7.12)	6.76 (6.19–7.39)	5.87 (5.65–6.11)	<0.001
**Switching to second‐line ART, *n* (%)**	490 (4)	292 (7)	182 (5)	964 (5)	<0.001
**Crude switching rate per 100 PY (95% CI)**	1.82 (1.67–1.99)	2.82 (2.52–3.16)	2.41 (2.09–2.79)	2.15 (2.02–2.29)	<0.001
**Duration from ART initiation to switching regimen, years**	1.51 (0.9–2.59)	1.52 (0.87–2.57)	1.45 (0.81–2.36)	1.5 (0.88–2.57)	0.75

Abbreviations: 95% CI, 95% confidence interval; LTFU, loss to follow‐up; PY, person‐year.

**^a^** The proportion was divided by the number of patients who had VL from 6 to 12 months after ART initiation.

### 3.3 Virological treatment outcomes

Of 19,825 patients starting ART, 17,512 (88%) had VL measurements from 6 to 12 months after starting ART. The rapid ART group had the highest proportion of patients with VL < 200 copies/ml (92%), followed by intermediate (89%) and delayed groups (88%; *p* < 0.001), respectively. Overall, 2512 (13%) experienced VF, a crude rate of 5.87 (95% CI 5.65–6.11) per 100 PY. The proportion with experienced VF on first‐line ART was 11% in the rapid ART group, and 15% in both the intermediate and delayed ART groups (*p* < 0.001). This equated to a crude VF rate in the rapid ART group of 5.35 (95% CI 2.13–2.66) per 100 PY, 6.59 (95% CI 6.11–7.12) per 100 PY in the intermediate ART and 6.76 (95% CI 6.19–7.39) per 100 PY in the delayed ART group (*p* < 0.001).

### 3.4 Factors associated with VF

Table 3 shows factors associated with VF on first‐line ART. In the multivariable model, VF was more frequent in the intermediate ART (adjusted incidence rate ratio, aIRR 1.14 [95% CI 1.03–1.25]) and delayed ART group (aIRR 1.47 [95% CI 1.33–1.63]) versus the rapid ART group. Females had a 96% (aIRR 1.96 [95% CI 1.80–2.13]) higher incidence rate of VF than males. YLHIV with current age < 20 years (aIRR 1.25, 95% CI 1.14–1.37) were more likely to have VF compared with those aged ≥ 20 years. YLHIV with pre‐ART CD4 counts <200 cells/mm^3^ (aIRR 2.77, 95% CI 2.51–3.06) and 200–350 cells/mm^3^ (aIRR 1.38, 95% CI 1.23–1.54) had higher VF rates versus those with CD4 counts ≥350 cells/mm^3^. Opportunistic infections before starting ART were associated with higher VF rates. Central, Northeastern and Western regions were significantly more likely to have VF compared to those in Bangkok.

**Table 3:** Incidence rate ratios for factors associated with virological failure (plasma HIV RNA ≥ 1000 copies/ml) after first‐line ART initiation

	**Univariable**		**Multivariable**	
**Characteristics**	**IRR (95% CI)**	***p***	**IRR (95% CI)**	***p***
**Gender**		<0.001		<0.001
Male	1 (ref)		1 (ref)	
Female	1.90 (1.75–2.06)		1.96 (1.80–2.13)	
**Current age (years)**		<0.001		<0.001
15 to < 20 years	1.21 (1.10–1.32)		1.25 (1.14–1.37)	
≥ 20 years	1 (ref)		1 (ref)	
**Rapid ART group**		<0.001		<0.001
Within 1 month	1 (ref)		1 (ref)	
>1–3 months	1.36 (1.24–1.50)		1.14 (1.03–1.25)	
>3 months	1.37 (1.23–1.52)		1.47 (1.33–1.63)	
**First regimen**		<0.001		<0.001
NVP based	1.73 (1.56–1.92)		1.48 (1.33–1.64)	
EVP based	1 (ref)		1 (ref)	
RPV based	0.30 (0.13–0.66)		0.40 (0.18–0.90)	
**Opportunistic infection at baseline**		<0.001		<0.001
Yes	1.97 (1.73–2.25)		1.31 (1.15–1.50)	
No	1 (ref)		1 (ref)	
**Year of ART initiation**		<0.001		0.20
2014–2016	1.21 (1.12–1.31)		1.07 (0.98–1.16)	
2017–2019	1 (ref)		1 (ref)	
**Pre‐ART CD4 count (cells/mm^3^)**		<0.001		<0.001
< 200	2.86 (2.54–3.22)		2.77 (2.51–3.06)	
200 to <350	1.12 (0.99–1.27)		1.38 (1.23–1.54)	
≥ 350	1 (ref)		1 (ref)	
Unknown	1.91 (1.57–2.32)		2.02 (1.68–2.41)	
**Region**		<0.001		0.025
Bangkok	1 (ref)		1 (ref)	
Central	1.36 (1.19–1.57)		1.18 (1.02–1.35)	
Northeastern	1.21 (1.06–1.38)		1.22 (1.07–1.39)	
Northern	1.31 (1.14–1.50)		1.07 (0.93–1.23)	
Eastern	1.41 (1.20–1.65)		1.14 (0.97–1.34)	
Southern	1.46 (1.23–1.72)		1.14 (0.96–1.35)	
Western	1.73 (1.41–2.13)		1.28 (1.04–1.58)	

Abbreviations: aIRR, adjusted incidence rate ratio; ART, antiretroviral therapy; 95% CI, 95% confidence interval; EFV, efavirenz; IRR, incidence rate ratio; NVP, nevirapine; ref, reference; RPV, rilpivirine.

### 3.5 Switch to second‐line ART regimen

**Figure 3:**
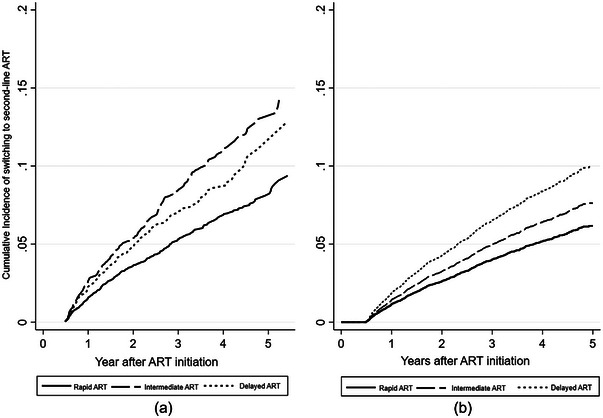
Cumulative incidence of switching to second‐line ART. Abbreviation: ART, antiretroviral therapy.

A total of 919 (5%) patients switched to second‐line PI‐based regimens (LPV/r 95%, ATV/r 4%, others 1%). The median duration from ART initiation to regimen switch was 1.50 (IQR 0.88–2.57) years. Second‐line switch rates were higher in YLHIV in the intermediate and delayed ART groups versus the rapid ART group (7% vs. 5% vs. 4%, *p*<0.001). The overall switch rate was 2.05 (95% CI 1.92–2.19) per 100 PY. The rate was lowest in the rapid ART group (1.70 [95% CI 1.55–1.86] per 100 PY) compared to other initiation groups. The cumulative incidence of second‐line ART switch at 1, 2 and 3 years in the rapid ART group was 1.6% (95% CI 1.3–1.8%), 3.6% (95% CI 3.2–4.0%) and 5.3% (95% CI 4.8–5.9%), respectively. For intermediate ART, the cumulative incidence of the second‐line switch at 1, 2 and 3 years was 2.7% (95% CI 2.2–3.2%), 5.4% (95% CI 4.6–6.2%) and 8.4% (95% CI 7.4–9.5%), respectively; and 2.3% (95% CI 1.8–2.9%), 4.8% (95% CI 4.0–5.7%) and 7.0% (95% CI 5.9–8.2%) in the delayed ART group, respectively (Figure 3a).

### 3.6 Factors associated with second‐line ART regimen

In the adjusted competing risk regression model (Table 4), YLHIV from delayed ART (adjusted sub‐hazard ratio, aSHR 1.65, 95% CI [1.38–1.97]) and intermediate ART (aSHR 1.25, 95% CI 1.07–1.45) were at higher risk of switching versus those in the rapid ART group (Figure 3b). Regimen switch rates were 53% higher in men (aSHR 1.53, 95% CI 1.32–1.77) than in women. Patients aged <20 years (aSHR 2.30 95% CI 1.92–2.76) were more likely to switch to second‐line ART compared to those aged ≥ 20 years. A lower pre‐ART CD4 count <200 cells/mm^3^ (aSHR 5.40, 95% CI 4.49–6.50) and pre‐ART CD4 count 200 to <350 cells/mm^3^ (aSHR 1.86, 95% CI 1.50–2.31) were at a higher risk of switching to second‐line regimens than those with CD4 counts ≥ 350 cells/mm^3^. YLHIV with opportunistic infections at baseline had a higher chance of switching to second‐line ART compared to those who did not. Regions and first regimen were also associated with the second‐line switch.

**Table 4:** Univariable and multivariable associations with switching to second‐line (PI‐based) ART from a competing risks regression model

Characteristics	Univariable		Multivariable	
	aSHR (95% CI)	*p*	aSHR (95% CI)	*p*
**Gender**		<0.001		<0.001
Male	1 (ref)		1 (ref)	
Female	1.63 (1.41–1.88)		1.53 (1.32–1.77)	
**Current age (years)**		<0.001		<0.001
15 to < 20 years	2.24 (1.87–2.68)		2.30 (1.92–2.76)	
≥ 20 years	1 (ref)		1 (ref)	
**Rapid ART group**		<0.001		<0.001
Within 1 month	1 (ref)		1 (ref)	
>1–3 months	1.62 (1.40–1.88)		1.25 (1.07–1.45)	
>3 months	1.35 (1.14–1.61)		1.65 (1.38–1.97)	
**First regimen**		<0.001		<0.001
NVP based	1.90 (1.62–2.21)		1.45 (1.23–1.70)	
EVP based	1 (ref)		1 (ref)	
RPV based	0.20 (0.03–1.46)		0.31 (0.04–2.24)	
**History of opportunistic infection at baseline**		<0.001		0.01
Yes	2.20 (1.81–2.67)		1.24 (1.01–1.52)	
No	1 (ref)		1 (ref)	
**Year of ART initiation**		0.56		
2014–2016	1.05 (0.90–1.22)			
2017–2019	1 (ref)			
**Pre‐ART CD4 count (cells/mm^3^)**		<0.001		<0.001
< 200	5.66 (4.75–6.74)		5.40 (4.49–6.50)	
200 to <350	1.81 (1.46–2.24)		1.86 (1.50–2.31)	
≥ 350	1 (ref)		1 (ref)	
Unknown	1.90 (1.35–2.69)		2.05 (1.45–2.90)	
**Region**		<0.001		0.03
Bangkok	1 (ref)		1 (ref)	
Central	1.49 (1.17–1.92)		1.19 (0.93–1.53)	
Northeastern	1.70 (1.35–2.14)		1.19 (0.94–1.51)	
Northern	1.64 (1.29–2.07)		1.17 (0.92–1.49)	
Eastern	2.08 (1.59–2.71)		1.44 (1.10–1.90)	
Southern	2.30 (1.76–3.01)		1.56 (1.19–2.05)	
Western	2.23 (1.58–3.15)		1.45 (1.01–2.07)	

Abbreviations: ART, antiretroviral therapy; aSHR, adjusted sub‐distribution hazard ratio; 95% CI, 95% confidence interval; EFV, efavirenz; NVP, nevirapine; ref, reference; RPV, rilpivirine; SHR, sub‐distribution hazard ratio.

Sensitivity analyses included only YLHIV (73%, *n* = 14,554) with an ascertained date of HIV diagnosis (Tables S1–S3). Rapid, intermediate and delayed ART initiation groups comprised 8945 (62%), 3236 (22%) and 2373 (16%, *p*<0.001) YLHIV, respectively. The median duration from HIV diagnosis to ART initiation was 22 (8–55) days. Outcomes after ART initiation were consistent with the main study outcomes, with an increased risk of VF second‐line ART switch in the intermediate and delayed ART, compared to the rapid ART group (Figure S1).

## 4 DISCUSSION

This is the first study assessing long‐term treatment outcomes among YLHIV in the Thai NAP, by timing of ART initiation after the “treat at any CD4 count” policy was adopted. Mortality of YLHIV starting rapid ART reduced with time, while the LTFU rates were similar among ART initiation groups. Viral suppression in the first year of treatment was also significantly higher among YLHIV in the rapid initiation group. Moreover, YLHIV starting rapid ART were less likely to have VF or switch to second‐ART, compared with those starting immediate or delayed ART. Similar results were found in sensitivity analyses which excluded those in whom the exact date of HIV diagnosis could not be ascertained.

These findings are likely effects of guideline changes with rapid ART initiation leading to better long‐term outcomes for YLHIV starting first‐line therapy. Moreover, uptake of rapid ART increased over time, as did pre‐ART CD4 in this group, whereas pre‐ART CD4 in the intermediate and delayed ART groups changed only slightly, perhaps partly because opportunistic infections as tuberculosis needed to be treated for 2–8 weeks before ART initiation [26].

Increases in rapid ART initiation observed after the implementation of a treat all policy in this current study are similar to a report from sub‐Saharan Africa, where a treat all policy adoption also increased the numbers of young adolescents accessing ART rapidly [4, 10]. Policy changes regarding rapid ART initiation led to decreased mortality rates and increased retention in care, similar to those in our study [29, 30]. YLHIV starting rapid ART had the lowest mortality rates, consistent with results from randomized control trials and cohort studies [27, 31]. Rapid ART initiation also accelerated viral suppression rates. Viral suppression at VL < 200 copies/ml increased from 75% in 2014–2018 to 82% in our second study period. This is consistent with studies from Egypt, Haiti and Taiwan where those who started rapid ART had increased rates of viral suppression [7, 31, 32, 33]. Socio‐economic and psychosocial factors can influence the timing of ART initiation and treatment outcomes. Previous studies from South Africa reported significant associations between timing of ART initiation and factors including level of facility, number of people in the household and having a partner [24, 34, 35].

Annual VL testing after ART initiation is offered for monitoring treatment in Thai YLHIV. Our analysis showed that VF rates were lowest in the rapid treatment group and highest in those initiating ART between 1–3 months, and >3 months after diagnosis. A study from 26 countries demonstrated that regular VL monitoring in adolescents has increased in low/lower‐middle‐income countries after the adoption of treat all policies, and is critical for the early detection of treatment failure [36, 37, 38]. Furthermore, YLHIV who started ART rapidly had a lower risk of switching to second‐line ART compared to other ART initiation groups. Ninety percent of switches to second‐line ART in our study had confirmed VF, with a median time to switch of 1.5 years. Our finding indicates a lower cumulative incidence of switching to second‐line ART after 3 years of rapid ART initiation (5.3%) compared to an individual patient data meta‐analysis study from Thailand (8%) and Europe (14–16%) before the era of treating at any CD4 levels [39]. Moreover, the proportion with confirmed VF with switching to second‐line ART in our study was also higher than adults living with HIV where rates ranged from 43% to 66% in studies from Eswatini, Uganda and Lesotho [40, 41, 42]. However, YLHIV aged < 20 years were at higher risk of switching to second‐line ART, compared to those aged older than 20 years [42, 43]. This finding suggests the scale‐up of rapid ART initiation in YLHIV would probably increase the rate of viral suppression and immediately switching to second‐line ART after detecting VF.

A strength of this study is the description of long‐term outcomes after rapid ART initiation in Thai YLHIV in a large National database, and the first study to assess long‐term treatment outcomes in Asian YLHIV by timing of ART initiation. It reflects real‐life practice and outcomes in a low‐middle income country in response to National treatment guideline changes, and demonstrates progress towards achieving the UNAIDS 95:95:95 goals. There are a number of limitations of this study. First, we defined the date of registration with NAP as a surrogate for the date of HIV diagnosis, which could create bias by shortening the time from HIV diagnosis to ART initiation, as some patients (37%) may have been diagnosed at private clinics and re‐tested at NAP registration. However, excluding these unknown diagnosis dates in a sensitivity analysis did not change the main study findings (Tables S1–S3). Second, the NAP database does not record details about socio‐economic and psychosocial factors, such as caregiver status, education, occupation, income and behavioural risks, so we could not examine the effects of these factors which might also influence access to rapid ART, as well as treatment outcomes. Third, 5% of YLHIV had missing pre‐ART CD4 counts, most likely because of delayed data entry or human error; however, we classified participants in this group as unknown CD4. Fourth, due to the observational nature of our study, the possibility of unobserved confounding influencing the relationship between ART initiation and study outcomes cannot be discounted. Last, this study was conducted from 2014 to 2019, prior to dolutegravir (DTG) implementation. It is, therefore, likely that rates of VF described in this study may continue to fall in the future, compared to YLHIV who initiated NNRTI‐based regimens in this study.

## 5 CONCLUSIONS

The proportion of YLHIV starting ART <30 days after HIV diagnosis significantly increased from 2014 to 2019 in the Thai NAP. These rapid ART initiators had a significantly lower risk of VF compared to those where initiation was delayed to 1–3, or >3 months. However, despite increasing rapid ART rates with predominantly EFV‐based regimens, VL suppression was below the 95% UNAIDS target. DTG rollout should be expedited to achieve better programme outcomes.

## COMPETING INTERESTS

KR has received the Senior Research Scholar from Thailand Research Fund (TRF). And he received honoraria or consultation fees from Merck, Roche, Jensen‐Cilag, Tibotec, Mylan and GPO (Governmental pharmaceutical organization, Thailand). He also has participated in a company‐sponsored speaker's bureau from Abbott, Gilead, Bristol‐Myers Squibb, Merck, Roche, Jensen‐Cilag, GlaxoSmithKline and GPO (Governmental pharmaceutical organization). TP received a clinical research grant from ViiV and MSD. ST was funded as a CIPHER grantee from the International AIDS Society, 2018–2020.

## AUTHORS’ CONTRIBUTIONS

ST, SJK, KR and TP created the study concept and study design. ST, SJK, PK and KR were responsible for data collection or oversaw programme implementation. ST conducted the analysis. SJK, KR and TP advised on the analysis. ST, SJK, PK, KR and TP interpreted the data. ST drafted the manuscript. All authors critically reviewed the manuscript and approved the manuscript for submission.

## FUNDING

The CIPHER grant was funded by the International AIDS Society, 2018–2020. HIV‐NAT, Thai Red Cross AIDS Research Centre, Bangkok, Thailand. International SciKU Branding (ISB) is funded by the Faculty of Science, Kasetsart University.

## DISCLAIMER

The content of this publication is solely the responsibility of the authors and does not necessarily represent the official views of any of the governments or institutions mentioned above. Data were presented as an oral presentation in part at the International Workshop on HIV Pediatrics 2021. Virtual conference, 16/07/2021–17/07/2021.

## Supporting information


**Table S1**: Characteristics of youth living with HIV at ART initiation stratified by time of ART initiation which limited to YLHIV with documented date of HIV diagnosis (n=14,554).
**Table S2**: Incidence rate ratios for factors associated with virological failure (plasma HIV RNA ≥ 1,000 copies/mL) after first‐line ART initiation (n=14,554).
**Table S3**: Univariable and multivariable associations with switching to second‐line (PI‐based) ART from a competing risks regression model (n=14,554).
**Figure S1**: Study outcomes after ART initiation in youth living with HIV in the Thai National HIV Treatment program, by timing of ART initiation (n=14,554).

## Data Availability

The data that support the findings of this study are available from the corresponding author upon reasonable request.

## References

[1] WHO . Guideline on when to start antiretroviral therapy and on pre‐exposure prophylaxis for HIV. World Health Organization; 2015. 26598776

[2] Tymejczyk O , Brazier E , Yiannoutsos CT , Vinikoor M , van Lettow M , Nalugoda F , et al. Changes in rapid HIV treatment initiation after national “treat all” policy adoption in 6 sub‐Saharan African countries: regression discontinuity analysis. PLoS Med. 2019; 16(6):e1002822. 31181056 10.1371/journal.pmed.1002822 PMC6557472

[3] Esber AL , Coakley P , Ake JA , Bahemana E , Adamu Y , Kiweewa F , et al. Decreasing time to antiretroviral therapy initiation after HIV diagnosis in a clinic‐based observational cohort study in four African countries. J Int AIDS Soc. 2020; 23(2):e25446. 32064776 10.1002/jia2.25446 PMC7025087

[4] Tymejczyk O , Brazier E , Yiannoutsos C , Wools‐Kaloustian K , Althoff K , Crabtree‐Ramirez B , et al. HIV treatment eligibility expansion and timely antiretroviral treatment initiation following enrollment in HIV care: a metaregression analysis of programmatic data from 22 countries. PLoS Med. 2018; 15(3):e1002534. 29570723 10.1371/journal.pmed.1002534 PMC5865713

[5] Ford N , Migone C , Calmy A , Kerschberger B , Kanters S , Nsanzimana S , et al. Benefits and risks of rapid initiation of antiretroviral therapy. AIDS. 2018; 32(1):17–23. 29112073 10.1097/QAD.0000000000001671 PMC5732637

[6] Lilian RR , Rees K , McIntyre JA , Struthers HE , Peters RPH . Same‐day antiretroviral therapy initiation for HIV‐infected adults in South Africa: analysis of routine data. PLoS One. 2020; 15(1):e0227572. 31935240 10.1371/journal.pone.0227572 PMC6959580

[7] Patel ND , Dallas RH , Knapp KM , Flynn PM , Gaur AH . Rapid start of antiretroviral therapy in youth diagnosed with HIV infection. Pediatr Infect Dis J. 2021; 40(2):147–50. 33399434 10.1097/INF.0000000000002969

[8] Seekaew P , Phanuphak N , Teeratakulpisarn N , Amatavete S , Lujintanon S , Teeratakulpisarn S , et al. Same‐day antiretroviral therapy initiation hub model at the Thai Red Cross Anonymous Clinic in Bangkok, Thailand: an observational cohort study. J Int AIDS Soc. 2021; 24(12):e25869. 34967504 10.1002/jia2.25869 PMC8717692

[9] WHO . Guidelines for managing advanced HIV disease and rapid initiation of antiretroviral therapy. Geneva; 2017. 29341560

[10] Tymejczyk O , Brazier E , Wools‐Kaloustian K , Davies MA , Dilorenzo M , Edmonds A , et al. Impact of universal antiretroviral treatment eligibility on rapid treatment initiation among young adolescents with human immunodeficiency virus in sub‐Saharan Africa. J Infect Dis. 2020; 222(5):755–64. 31682261 10.1093/infdis/jiz547 PMC7530553

[11] Seekaew P , Pengnonyang S , Jantarapakde J , Sungsing T , Rodbumrung P , Trachunthong D , et al. Characteristics and HIV epidemiologic profiles of men who have sex with men and transgender women in key population‐led test and treat cohorts in Thailand. PLoS One. 2018; 13(8):e0203294. 30161226 10.1371/journal.pone.0203294 PMC6117046

[12] van Griensven F , Phanuphak N , Manopaiboon C , Dunne EF , Colby DJ , Chaiphosri P , et al. HIV prevalence and incidence among men who have sex with men and transgender women in Bangkok, 2014–2018: outcomes of a consensus development initiative. PLoS One. 2022; 17(1):e0262694. 35061803 10.1371/journal.pone.0262694 PMC8782340

[13] Teeraananchai S , Puthanakit T , Kerr SJ , Chaivooth S , Kiertiburanakul S , Chokephaibulkit K , et al. Attrition and treatment outcomes among adolescents and youths living with HIV in the Thai National AIDS Program. J Virus Erad. 2019; 5(1):33–40. 30800424 10.1016/S2055-6640(20)30276-4 PMC6362904

[14] Teeraananchai S , Kerr SJ , Ruxrungtham K , Avihingsanon A , Chaivooth S , Teeraratkul A , et al. Loss to follow‐up and associated factors of patients in the National AIDS Program in Thailand. Antivir Ther. 2018; 23(6):529–38. 29583122 10.3851/IMP3233

[15] Agolory SG , Auld AF , Odafe S , Shiraishi RW , Dokubo EK , Swaminathan M , et al. High rates of loss to follow‐up during the first year of pre‐antiretroviral therapy for HIV patients at sites providing pre‐ART care in Nigeria, 2004–2012. PLoS One. 2017; 12(9):e0183823. 28863160 10.1371/journal.pone.0183823 PMC5581182

[16] Tweya H , Oboho IK , Gugsa ST , Phiri S , Rambiki E , Banda R , et al. Loss to follow‐up before and after initiation of antiretroviral therapy in HIV facilities in Lilongwe, Malawi. PLoS One. 2018; 13(1):e0188488. 29373574 10.1371/journal.pone.0188488 PMC5786288

[17] Fox MP , Shearer K , Maskew M , Meyer‐Rath G , Clouse K , Sanne I . Attrition through multiple stages of pre‐treatment and ART HIV care in South Africa. PLoS One. 2014; 9(10):e110252. 25330087 10.1371/journal.pone.0110252 PMC4203772

[18] Evangeli M , Newell ML , McGrath N . Factors associated with pre‐ART loss‐to‐follow up in adults in rural KwaZulu‐Natal, South Africa: a prospective cohort study. BMC Public Health. 2016; 16:358. 27117271 10.1186/s12889-016-3025-x PMC4847371

[19] Bor J , Fox MP , Rosen S , Venkataramani A , Tanser F , Pillay D , et al. Treatment eligibility and retention in clinical HIV care: a regression discontinuity study in South Africa. PLoS Med. 2017; 14(11):e1002463. 29182641 10.1371/journal.pmed.1002463 PMC5705070

[20] Khan S , Spiegelman D , Walsh F , Mazibuko S , Pasipamire M , Chai B , et al. Early access to antiretroviral therapy versus standard of care among HIV‐positive participants in Eswatini in the public health sector: the MaxART stepped‐wedge randomized controlled trial. J Int AIDS Soc. 2020; 23(9):e25610. 32949103 10.1002/jia2.25610 PMC7507004

[21] Bantie B , Abate MW , Nigat AB , Birlie TA , Dires T , Minuye T , et al. Attrition rate and its predictors among adults receiving anti‐retroviral therapy following the implementation of the “Universal Test and Treat strategy” at public health institutions in Northern Ethiopia. A retrospective follow‐up study. Heliyon. 2022; 8(11):e11527. 36411907 10.1016/j.heliyon.2022.e11527 PMC9674913

[22] Bantie B , Seid A , Kerebeh G , Alebel A , Dessie G . Loss to follow‐up in “test and treat era” and its predictors among HIV‐positive adults receiving ART in Northwest Ethiopia: institution‐based cohort study. Front Public Health. 2022; 10:876430. 36249247 10.3389/fpubh.2022.876430 PMC9557930

[23] Alhaj M , Amberbir A , Singogo E , Banda V , van Lettow M , Matengeni A , et al. Retention on antiretroviral therapy during Universal Test and Treat implementation in Zomba district, Malawi: a retrospective cohort study. J Int AIDS Soc. 2019; 22(2):e25239. 30734510 10.1002/jia2.25239 PMC6367572

[24] Ross J , Brazier E , Fatti G , Jaquet A , Tanon A , Haas AD , et al. Same‐day ART initiation as a predictor of loss to follow‐up and viral suppression among people living with HIV in sub‐Saharan Africa. Clin Infect Dis. 2023;76(1):39–47. 36097726 10.1093/cid/ciac759 PMC10202422

[25] Chaivooth S , Bhakeecheep S , Ruxrungtham K , Teeraananchai S , Kerr SJ , Teeraratkul A , et al. The challenges of ending AIDS in Asia: outcomes of the Thai National AIDS Universal Coverage Programme, 2000–2014. J Virus Erad. 2017; 3(4):192–9. 29057081 10.1016/S2055-6640(20)30323-X PMC5632544

[26] Manosuthi W , Ongwandee S , Bhakeecheep S , Leechawengwongs M , Ruxrungtham K , Phanuphak P , et al. Guidelines for antiretroviral therapy in HIV‐1 infected adults and adolescents 2014, Thailand. AIDS Res Ther. 2015; 12:12. 25908935 10.1186/s12981-015-0053-z PMC4407333

[27] Teeraananchai S , Kerr SJ , Khananuraksa P , Ruxrungtham K , Puthanakit T . Rapid antiretroviral initiation among Thai youth living with HIV in the National AIDS programme in the era of treatment at any CD4 cell count: a national registry database study. J Int AIDS Soc. 2020; 23(Suppl 5):e25574. 32869537 10.1002/jia2.25574 PMC7459169

[28] Fine JP , Gray RJ . A proportional hazards model for the sub distribution of a competing risk. J Am Statist Assoc. 1999; 94:496–509.

[29] Makurumidze R , Buyze J , Decroo T , Lynen L , de Rooij M , Mataranyika T , et al. Patient‐mix, programmatic characteristics, retention and predictors of attrition among patients starting antiretroviral therapy (ART) before and after the implementation of HIV “Treat All” in Zimbabwe. PLoS One. 2020; 15(10):e0240865. 33075094 10.1371/journal.pone.0240865 PMC7571688

[30] Mody A , Sikazwe I , Czaicki NL , Wa Mwanza M , Savory T , Sikombe K , et al. Estimating the real‐world effects of expanding antiretroviral treatment eligibility: evidence from a regression discontinuity analysis in Zambia. PLoS Med. 2018; 15(6):e1002574. 29870531 10.1371/journal.pmed.1002574 PMC5988277

[31] Koenig SP , Dorvil N , Devieux JG , Hedt‐Gauthier BL , Riviere C , Faustin M , et al. Same‐day HIV testing with initiation of antiretroviral therapy versus standard care for persons living with HIV: a randomized unblinded trial. PLoS Med. 2017; 14(7):e1002357. 28742880 10.1371/journal.pmed.1002357 PMC5526526

[32] Huang YC , Sun HY , Chuang YC , Huang YS , Lin KY , Huang SH , et al. Short‐term outcomes of rapid initiation of antiretroviral therapy among HIV‐positive patients: real‐world experience from a single‐centre retrospective cohort in Taiwan. BMJ Open. 2019; 9(9):e033246. 10.1136/bmjopen-2019-033246 PMC6756335 31542770

[33] Bacon O , Chin J , Cohen SE , Hessol NA , Sachdev D , Coffey S , et al. Decreased time from human immunodeficiency virus diagnosis to care, antiretroviral therapy initiation, and virologic suppression during the citywide RAPID initiative in San Francisco. Clin Infect Dis. 2021; 73(1):e122–8. 32449916 10.1093/cid/ciaa620 PMC8561247

[34] Onoya D , Sineke T , Hendrickson C , Mokhele I , Maskew M , Long LC , et al. Impact of the test and treat policy on delays in antiretroviral therapy initiation among adult HIV positive patients from six clinics in Johannesburg, South Africa: results from a prospective cohort study. BMJ Open. 2020; 10(3):e030228. 10.1136/bmjopen-2019-030228 PMC7170559 32213514

[35] Boyer S , Iwuji C , Gosset A , Protopopescu C , Okesola N , Plazy M , et al. Factors associated with antiretroviral treatment initiation amongst HIV‐positive individuals linked to care within a universal test and treat programme: early findings of the ANRS 12249 TasP trial in rural South Africa. AIDS Care. 2016; 28(Suppl 3):39–51. 27421051 10.1080/09540121.2016.1164808 PMC5096681

[36] Brazier E , Tymejczyk O , Zaniewski E , Egger M , Wools‐Kaloustian K , Yiannoutsos CT , et al. Effects of national adoption of treat‐all guidelines on pre‐antiretroviral therapy (ART) CD4 testing and viral load monitoring after ART initiation: a regression discontinuity analysis. Clin Infect Dis. 2021; 73(6):e1273–81. 33693517 10.1093/cid/ciab222 PMC8442775

[37] Kibalama Ssemambo P , Nalubega‐Mboowa MG , Owora A , Serunjogi R , Kironde S , Nakabuye S , et al. Virologic response of treatment experienced HIV‐infected Ugandan children and adolescents on NNRTI based first‐line regimen, previously monitored without viral load. BMC Pediatr. 2021; 21(1):139. 33752636 10.1186/s12887-021-02608-0 PMC7983217

[38] Mesic A , Spina A , Mar HT , Thit P , Decroo T , Lenglet A , et al. Predictors of virological failure among people living with HIV receiving first line antiretroviral treatment in Myanmar: retrospective cohort analysis. AIDS Res Ther. 2021; 18(1):16. 33882962 10.1186/s12981-021-00336-0 PMC8059266

[39] European Pregnancy and Paediatric HIV Cohort Collaboration . Time to switch to second‐line antiretroviral therapy in children with human immunodeficiency virus in Europe and Thailand. Clin Infect Dis. 2018; 66(4):594–603. 29029056 10.1093/cid/cix854 PMC5796645

[40] Etoori D , Ciglenecki I , Ndlangamandla M , Edwards CG , Jobanputra K , Pasipamire M , et al. Successes and challenges in optimizing the viral load cascade to improve antiretroviral therapy adherence and rationalize second‐line switches in Swaziland. J Int AIDS Soc. 2018; 21(10):e25194. 30350392 10.1002/jia2.25194 PMC6198167

[41] Glass TR , Motaboli L , Nsakala B , Lerotholi M , Vanobberghen F , Amstutz A , et al. The viral load monitoring cascade in a resource‐limited setting: a prospective multicentre cohort study after introduction of routine viral load monitoring in rural Lesotho. PLoS One. 2019; 14(8):e0220337. 31461455 10.1371/journal.pone.0220337 PMC6713472

[42] Ssempijja V , Nakigozi G , Chang L , Gray R , Wawer M , Ndyanabo A , et al. Rates of switching to second‐line antiretroviral therapy and impact of delayed switching on immunologic, virologic, and mortality outcomes among HIV‐infected adults with virologic failure in Rakai, Uganda. BMC Infect Dis. 2017; 17(1):582. 28830382 10.1186/s12879-017-2680-6 PMC5568262

[43] Kyaw NT , Harries AD , Kumar AM , Oo MM , Kyaw KW , Win T , et al. High rate of virological failure and low rate of switching to second‐line treatment among adolescents and adults living with HIV on first‐line ART in Myanmar, 2005–2015. PLoS One. 2017; 12(2):e0171780. 28182786 10.1371/journal.pone.0171780 PMC5300167

