# Supplementary material for: Long‐term outcomes of rapid antiretroviral NNRTI‐based initiation among Thai youth living with HIV: a national registry database study
Source: J Int AIDS Soc. 2023 Mar 21;26(3):e26071. doi: 10.1002/jia2.26071 (PMC10029993; doi:10.1002/jia2.26071)
Supplement: Supplementary file 1 — Table S1: Characteristics of youth living with HIV at ART initiation stratified by time of ART initiation which limited to YLHIV with documented date of HIV diagnosis (n=14,554). Table S2: Incidence rate ratios for factors associated with virological failure (plasma HIV RNA ≥ 1,000 copies/mL) after first‐line ART initiation (n=14,554). Table S3: Univariable and multivariable associations with switching to second‐line (PI‐based) ART from a competing risks regression model (n=14,554). Figure S1: Study outcomes after ART initiation in youth living with HIV in the Thai National HIV Treatment program, by timing of ART initiation (n=14,554). [file JIA2-26-e26071-s001.docx]

**Supplementary Table 1 Characteristics of youth living with HIV at ART initiation stratified by time of ART initiation which limited to YLHIV with documented date of HIV diagnosis (n=14,554).**

| **Characteristics** | **ART initiation group (Duration from Diagnosis to starting ART)** | | | | **P** |
| --- | --- | --- | --- | --- | --- |
|  | **Rapid** | **Intermediate** | **Delayed** | **Total** |  |
|  | **(<1 month)** | **(1-3months)** | **(>3months)** |  |  |
| **N (%)** | 8,945 (61) | 3,236 (22) | 2,373 (16) | 14,554 |  |
| **Gender, N (%)** |  |  |  |  |  |
| Male | 6,748 (75) | 2,646 (82) | 1,959 (83) | 11,353 (78) | <0.001 |
| Female | 2,197 (25) | 590 (18) | 414 (17) | 3,201 (22) |  |
| **Median (IQR) age (years)** | 21 (19-23) | 21 (20-23) | 22 (20-24) | 21 (20-23) | <0.001 |
| **Age category** |  |  |  |  | <0.001 |
| 15-<20 years | 2,998 (34) | 957 (30) | 462 (19) | 4,417 (30) |  |
| ≥20 years | 5,947 (66) | 2,279 (70) | 1,911 (81) | 10,137 (70) |  |
| **Pre-ART CD4 available, N (%)** | 8,458 (95) | 3,138 (97) | 2,194 (92) | 13,790 (95) |  |
| **Median (IQR) Pre-ART CD4 count (cells/mm^3^)** | 347 (214-500) | 277 (96-445) | 390 (208-557) | 339 (189-498) | <0.001 |
| **First regimen** |  |  |  |  | <0.001 |
| NVP based | 511 (6) | 375 (12) | 211 (9) | 1,097 (8) |  |
| EVP based | 8,343 (93) | 2,839 (88) | 2,120 (89) | 13,302 (91) |  |
| RPV based | 91 (1) | 22 (1) | 42 (2) | 155 (1) |  |
| **History of Opportunistic infection** |  |  |  |  | <0.001 |
| Yes | 264 (3) | 291 (9) | 173 (7) | 728 (5) |  |
| No | 8,681 (97) | 2,945 (91) | 2,200 (93) | 13,826 (95) |  |
| **Year of ART initiation** |  |  |  |  | <0.001 |
| 2014-2016 | 2,981 (33) | 1,354 (42) | 897 (38) | 5,232 (36) |  |
| 2017-2019 | 5,964 (67) | 1,882 (58) | 1,476 (62) | 9,322 (64) |  |
| **Region** |  |  |  |  | <0.001 |
| Bangkok | 1,670 (19) | 349 (11) | 452 (19) | 2,471 (17) |  |
| Central | 1,387 (16) | 576 (18) | 342 (14) | 2,305 (16) |  |
| North-eastern | 2,441 (27) | 906 (28) | 535 (23) | 3,882 (27) |  |
| Northern | 1,798 (20) | 586 (18) | 411 (17) | 2,795 (19) |  |
| Eastern | 783 (9) | 309 (10) | 280 (12) | 1,372 (9) |  |
| Southern | 637 (7) | 319 (10) | 221 (9) | 1,177 (8) |  |
| Western | 229 (3) | 191 (6) | 132 (6) | 552 (4) |  |

Abbreviations: ART, antiretroviral therapy; NVP-Nevirapine; EFV- Efavirenz; RPV- [Rilpivirine](https://clinicalinfo.hiv.gov/en/guidelines/perinatal/rilpivirine-edurant-rpv)

Presented as n (%) for categorical data and median (interquartile range) for continuous data.

The comparisons were performed using Pearson’s Chi-square tests, for categorical data, and Kruskal-wallis tests for continuous data.

Note: We included only youth living with HIV (73%, n=14,554/29,825) who had an ascertained date of HIV diagnosis to classify ART initiation group in sensitivity analyses.

**Supplementary Table 2 Incidence rate ratios for factors associated with virological failure (plasma HIV RNA ≥ 1,000 copies/mL) after first-line ART initiation (n=14,554).**

| **Characteristics** | **Univariable** | | **Multivariable** | |
| --- | --- | --- | --- | --- |
|  | **IRR (95%CI)** | **P** | **aIRR (95%CI)** | **P** |
| **Gender** |  | <0.001 |  | <0.001 |
| Male | 1 (ref) |  | 1 (ref) |  |
| Female | 2.00 (1.81-2.21) |  | 2.10 (1.90-2.32) |  |
| **Current Age (years)** |  | <0.001 |  | 0.004 |
| 15-< 20 years | 1.17 (1.05-1.31) |  | 1.19 (1.07-1.34) |  |
| ≥ 20 years | 1 (ref) |  | 1 (ref) |  |
| **Rapid ART group** |  | <0.001 |  | <0.001 |
| Within 1 month | 1 (ref) |  | 1 (ref) |  |
| >1-3months | 1.35 (1.21-1.52) |  | 1.14 (1.03-1.25) |  |
| >3months | 1.44 (1.27-1.63) |  | 1.47 (1.33-1.63) |  |
| **First regimen** |  | <0.001 |  | <0.001 |
| NVP based | 1.77 (1.55-2.03) |  | 1.52 (1.32-1.75) |  |
| EVP based | 1 (ref) |  | 1 (ref) |  |
| RPV based | 0.35 (0.15-0.82) |  | 0.47 (0.20-1.11) |  |
| **History of opportunistic infection at baseline** |  | <0.001 |  | <0.001 |
| Yes | 2.20 (1.89-2.56) |  | 1.43 (1.22-1.67) |  |
| No | 1 (ref) |  | 1 (ref) |  |
| **Year of ART initiation** |  | <0.001 |  | 0.69 |
| 2014-2016 | 1.19 (1.08-1.31) |  | 1.03 (0.93-1.14) |  |
| 2017-2019 | 1 (ref) |  | 1 (ref) |  |
| **Pre-ART CD4 count (cells/mm^3^)** |  | <0.001 |  | <0.001 |
| < 200 | 2.99 (2.67-3.35) |  | 2.83 (2.51-3.18) |  |
| 200-<350 | 1.33 (1.16-1.53) |  | 1.37 (1.19-1.57) |  |
| ≥ 350 | 1 (ref) |  | 1 (ref) |  |
| unknown | 1.95 (1.57-2.43) |  | 2.05 (1.65-2.55) |  |
| **Region** |  | <0.001 |  | 0.20 |
| Bangkok | 1 (ref) |  | 1 (ref) |  |
| Central | 1.5 (1.25-1.8) |  | 1.25 (1.04-1.50) |  |
| North-eastern | 1.65 (1.39-1.95) |  | 1.33 (1.12-1.57) |  |
| Northern | 1.43 (1.2-1.71) |  | 1.15 (0.96-1.37) |  |
| Eastern | 1.54 (1.25-1.89) |  | 1.19 (0.96-1.46) |  |
| Southern | 1.49 (1.2-1.85) |  | 1.10 (0.89-1.37) |  |
| Western | 2.05 (1.6-2.63) |  | 1.47 (1.15-1.88) |  |

IRR- incidence rate ratio; aIRR-adjusted incidence rate ratio, 95%CI-95%confidence interval, ref – reference.

Abbreviations: ART, antiretroviral therapy; NVP-Nevirapine; EFV- Efavirenz; RPV- Rilpivirine

Note: We included only youth living with HIV (73%, n=14,554/29,825) who had the ascertained date of HIV diagnosis to classify ART initiation group for sensitivity analysis.

**Supplementary Table 3 Univariable and multivariable associations with switching to second-line (PI-based) ART from a competing risks regression model (n=14,554).**

| **Characteristics** | **Univariable** | | **Multivariable** | |
| --- | --- | --- | --- | --- |
|  | **SHR (95%CI)** | **P** | **aSHR (95%CI)** | **P** |
| **Gender** |  | <0.001 |  | <0.001 |
| Male | 1 (ref) |  | 1 (ref) |  |
| Female | 1.76 (1.48-2.10) |  | 1.71 (1.43-2.04) |  |
| **Current Age (years)** |  | <0.001 |  | <0.001 |
| 15-< 20 years | 2.00 (1.60-2.49) |  | 2.07 (1.65-2.59) |  |
| ≥ 20 years | 1 (ref) |  | 1 (ref) |  |
| **Rapid ART group** |  | <0.001 |  | <0.001 |
| within1month | 1 (ref) |  | 1 (ref) |  |
| >1-3 months | 1.61 (1.34-1.93) |  | 1.20 (1.01-1.44) |  |
| >3 months | 1.41 (1.14-1.75) |  | 1.65 (1.32-2.06) |  |
| **First regimen** |  | <0.001 |  | <0.001 |
| NVP based | 1.91 (1.55-2.35) |  | 1.42 (1.15-1.76) |  |
| EVP based | 1 (ref) |  | 1 (ref) |  |
| RPV based | 0.28 (0.04-2.03) |  | 0.44 (0.06-3.11) |  |
| **History of Opportunistic infection at baseline** |  | <0.001 |  | 0.001 |
| Yes | 2.64 (2.09-3.33) |  | 1.42 (1.11-1.81) |  |
| No | 1 (ref) |  | 1 (ref) |  |
| **Year of ART initiation** |  | 0.35 |  |  |
| 2014-2016 | 1.09 (0.91-1.31) |  |  |  |
| 2017-2019 | 1 (ref) |  |  |  |
| **Pre-ART CD4 count (cells/mm^3^)** |  | <0.001 |  | <0.001 |
| < 200 | 5.76 (4.64-7.14) |  | 5.5 (4.37-6.93) |  |
| 200-<350 | 1.77 (1.35-2.31) |  | 1.84 (1.4-2.41) |  |
| ≥ 350 | 1 (ref) |  | 1 (ref) |  |
| unknown | 1.52 (0.93-2.46) |  | 1.60 (0.98-2.60) |  |
| **Region** |  | <0.001 |  | 0.14 |
| Bangkok | 1 (ref) |  | 1 (ref) |  |
| Central | 1.42 (1.02-1.98) |  | 1.04 (0.74-1.45) |  |
| North-eastern | 1.64 (1.21-2.22) |  | 1.06 (0.78-1.45) |  |
| Northern | 1.61 (1.18-2.20) |  | 1.06 (0.77-1.45) |  |
| Eastern | 1.98 (1.39-2.82) |  | 1.27 (0.89-1.82) |  |
| Southern | 2.21 (1.56-3.12) |  | 1.30 (0.91-1.86) |  |
| Western | 2.49 (1.64-3.79) |  | 1.53 (0.99-2.35) |  |

SHR- sub-distribution hazard ratio; aSHR-adjusted sub-distribution hazard ratio, 95%CI-95%confidence interval, ref – reference.

Abbreviations: ART, antiretroviral therapy; NVP-Nevirapine; EFV- Efavirenz; RPV- [Rilpivirine](https://clinicalinfo.hiv.gov/en/guidelines/perinatal/rilpivirine-edurant-rpv)

[Note: We included only youth living with HIV (73%, n=14,554/29,825) who had the ascertained date of HIV diagnosis to classify ART initiation group for sensitivity analysis.](https://clinicalinfo.hiv.gov/en/guidelines/perinatal/rilpivirine-edurant-rpv)

**[Supplementary Figure 1 Study outcomes after ART initiation in youth living with HIV in the Thai National HIV Treatment program, by timing of ART initiation (n=14,554).](https://clinicalinfo.hiv.gov/en/guidelines/perinatal/rilpivirine-edurant-rpv)**

[Abbreviations: ART, antiretroviral therapy](https://clinicalinfo.hiv.gov/en/guidelines/perinatal/rilpivirine-edurant-rpv)

Note: We included only youth living with HIV (73%, n=14,554/29,825) who had the ascertained date of HIV diagnosis to classify ART initiation group for sensitivity analysis.

There were statistically significant differences in the proportion of youth experiencing all outcomes by ART initiation group (P < 0.001).
